# Regulatory T cells and co‐evolution of allele‐specific MHC recognition by the TCR

**DOI:** 10.1111/sji.12853

**Published:** 2019-12-17

**Authors:** Edward J. Steele, Robyn A. Lindley

**Affiliations:** ^1^ Melville Analytics Pty Ltd Melbourne Vic Australia; ^2^ CYO’Connor ERADE Village Foundation Perth WA Australia; ^3^ GMDxCo Pty Ltd Melbourne Vic Australia; ^4^ Department of Clinical Pathology Faculty of Medicine Dentistry & Health Sciences University of Melbourne Melbourne Vic Australia

**Keywords:** AID/APOBEC and ADAR deaminases, DNA polymerase‐η, Germline TCR V Repertoires, somatic hypermutation, soma‐to‐germline feedback, thymic and induced tregs

## Abstract

What is the evolutionary mechanism for the TCR‐MHC‐conserved interaction? We extend Dembic's model (Dembic Z. In, Scand J Immunol e12806, 2019) of thymus positive selection for high‐avidity anti–self‐MHC Tregs among double (CD4 + CD8+)‐positive (DP) developing thymocytes. This model is based on competition for self‐MHC (+ Pep) complexes presented on cortical epithelium. Such T cells exit as CD4 + CD25+FoxP3 + thymic‐derived Tregs (tTregs). The other positively selected DP T cells are then negatively selected on medulla epithelium removing high‐avidity anti–self‐MHC + Pep as T cells commit to CD4 + or CD8 + lineages. The process is likened to the competitive selection and affinity maturation in Germinal Centre for the somatic hypermutation (SHM) of rearranged immunoglobulin (Ig) variable region (V[D]Js) of centrocytes bearing antigen‐specific B cell receptors (BCR). We now argue that the *same direct* SHM processes for TCRs occur in post‐antigenic Germinal Centres, but now occurring in peripheral pTregs. This model provides a potential solution to a long‐standing problem previously recognized by Cohn and others (Cohn M, Anderson CC, Dembic Z. In, Scand J Immunol e12790, 2019) of *how* co‐evolution occurs of species‐specific MHC alleles with the repertoire of their germline TCR V counterparts. We suggest this is not by ‘blind’, slow, and random Darwinian natural selection events, but a rapid structured somatic selection vertical transmission process. The pTregs bearing somatic TCR V mutant genes then, on arrival in reproductive tissues, can donate their TCR V sequences via soma‐to‐germline feedback as discussed in this journal earlier. (Steele EJ, Lindley RA. In, Scand J Immunol e12670, 2018) The high‐avidity tTregs also participate in the same process to maintain a biased, high‐avidity anti–self‐MHC germline V repertoire.

## 1 INTRODUCTION

In this paper, we address the challenge of explaining how thymic and peripheral regulatory T cells work to ensure the co‐evolution of germline TCR V repertoires with species‐specific MHC alleles. This follows from our earlier discussion^1^ following Cohn^2^ on the evolution of the Stage I germline Ig V segment repertoire expressed in B1b‐like (B‐0) lymphocytes. These Ig V elements can be considered to largely encode VH and VL sequences for antibody specificities that are specific for predicable self‐antigens that appear in each ontogeny such as those determinants on buried, effete or aged self‐components. These specificities are the familiar background, often cross‐reactive, IgM ‘natural antibodies’ detected in normal healthy plasma in humans, mice and other vertebrates.^3^ We now extend the discussion on how thymic regulatory T (tTreg) cells work by Dembic^4^ and deal directly with the implications for understanding the mechanism of germline evolution of the allele‐specific TCR V segment repertoires.

To begin, our question is: Are Germinal Centre follicular Tregs (CD4+Tfregs, CD8+ Tfregs) the cellular site of somatic mutation and positive selection via AID/APOBEC‐deaminase induced (and ADAR1‐deaminase/DNA Polymerase‐η coupled) somatic hypermutation (SHM) of rearranged TCR V[D]J regions? If this is indeed the case we argue that such TCR mutations may occur ‘safely’ within Tregs without severe functional consequence. That is, they would occur within the context of the specific regulation of anti‐self *versus* anti–non‐self responses, while not maturing into more lethal and overt anti‐self effectors that may result in harmful autoimmunity. We join this idea with a reconsideration of earlier Germinal Centre TCR/SHM observations by work in immunized mice to non‐replicating hapten‐protein conjugates of Kelsoe and associates (mainly in rearranged Vα11 segments)^5^, and later confirmed in humans for HIV‐1 infection by the Paris group of Wain‐Hobson and associates (mainly in the rearranged VβV2 and VβV5 segments)^6^.

The next evolutionary step would then imply a mechanism for soma‐to‐germline feedback via these peripheral Tregs delivering such somatically GC‐selected TCR Vβ and Vα sequences to the germline TCR V arrays (as reviewed for B1b‐like (B‐0) lymphocytes.^1^ Such deaminase‐based mutation processes that are initiated in an innate immune response to an invading pathogen and then undergo positive somatic selection would work co‐operatively to conserve the V_CDR1+2_ higher affinity (avidity) for self‐MHC I/II (the species MHC alleles). The deaminase‐based mutational processes would also provide germline V region starting points for recognition of common epitopes on re‐current viral pathogens. Then, both sets of somatic V specificities could potentially be delivered to the germline V arrays and become embedded by retro‐gene conversion mechanisms. That is via soma‐to‐germline V feedback processes as previously proposed.^1^, ^7^, ^8^, ^9^, ^10^, ^11^, ^12^

It should be noted that this new explanation is analogous to the more readily understandable anti‐self germline V repertoires in B cell Ig V evolution,^1^ where such acquired inheritance events are envisaged to occur in each surviving parent prior to reproduction.^1^, ^7^, ^10^, ^11^, ^12^ This explanation is also consistent with the recent findings of Lindley and Hall^13^ showing that there is evidence that the inherited source of many SNPs arising in the human genome are likely to be the result of deaminase‐based uncorrected new somatic mutations such as one might acquire during an innate immune response to a human pathogen and affecting on many other non‐Ig genes. Given, as Dembic argues, that high‐avidity self‐MHCI/II binding will be restricted to tTregs, then it is these cells that form the starting point for the focus of our proposal here.

We believe that by co‐opting Dembic’s proposal into a somatic selection and soma‐to‐germline model, it helps resolve the long‐standing dual recognition, positive and negative selection contradictions, in Standard and Tritope models of TCR antigen recognition. The positively selected high‐affinity anti–self‐MHCI/II TCRs expanded and expressed in tTregs and pTregs via their passage through first the thymus, and then the Germinal Centres, can then potentially target reproductive tissues harbouring the germline V genes while patrolling around the body to maintain the integrity of immunity and self‐tolerance during pathogen infection, and other chronic diseases, such as endogenous tumours.^14^ In parallel to these processes, the normal peripheral T cell repertoire of CD4+ T helper and CD8+ T cytotoxic cells bear the less avid (milder) self‐MHC reactivity and behave as MHC restricted lineages responsive to foreign peptide antigens. Thus, conventional peripheral CD4+ and CD8+ expressed TCRs bind both a restrictive MHCI/II molecule with a peptide in the binding cleft (pMHC ligand), and thus allowing an immune activation signal to be transmitted into the T helper or T cytotoxic cells.^4^

## 2 WHY SHOULD THE TCR SOMATICALLY MUTATE?

**Figure 1:**
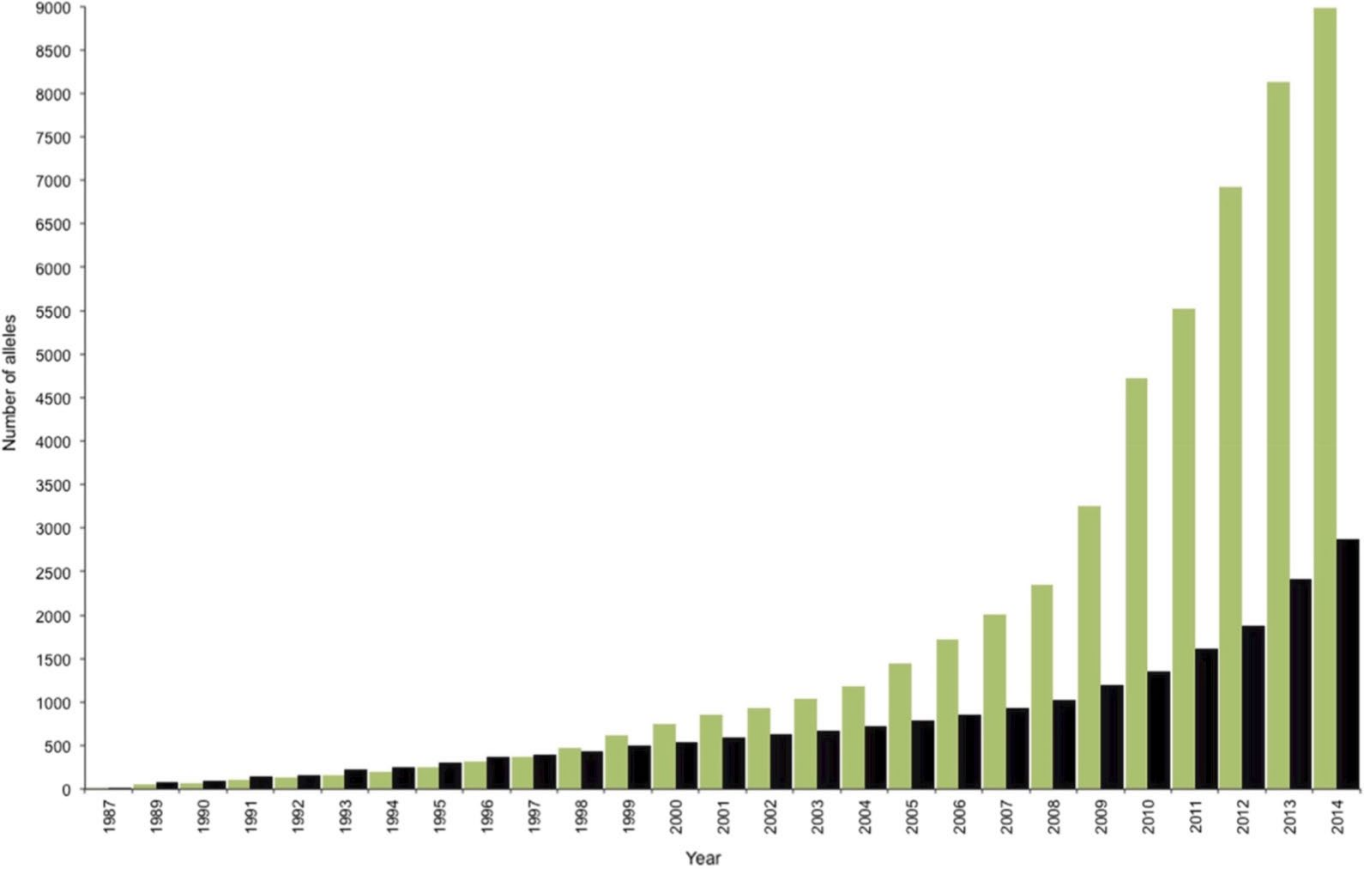
Growth of the IMGT/HLA Database. The number of allele sequences deposited annually in the IMGT/HLA Database is shown for class I (green), class II (black). The slope of the line reflects the rate of acquisition, which has accelerated in recent years. This is a copy of Figure 1 from the OPEN‐ACCESS article by Robinson et al^16^

The sceptic’s question however is this: Why should we ever need new germline TCR Vs that emerge by somatic hypermutation and soma‐to‐germline feedback? In our opinion, it is crucially important to ensure ‘germline tracking’ and thus co‐evolution of germline TCR V repertoires with repertoires of species‐specific MHCI/II alleles, which are *also rapidly evolving*, particularly in *Homo sapiens* see Parham and Ohta 1996^15^ cf. the high discovery rate of new human MHCI/II alleles, particularly MHC I alleles, and see the striking J‐curve histogram plot in Figure 1 (from Robinson et al 2015).^16^ That is, there is a sound argument for a requisite TCR V repertoire to germline track MHC alleles. It is maintained by somatic selection in an individual and helps maintain central tolerance against self T cell and B cell reactivities, and during evolution via an implied soma‐to‐germline feedback loop. In such a model, the germline V segment arrays are effectively updated and remain synchronized with the constantly changing species‐specific MHCI/II allele repertoire (Figure 1).^16^

These processes are also possibly accelerated by migration and inter‐ethnic matings.^15^ Thus underpinning the Parham and Ohta^15^ analyses are all those novel HLA alleles which appear to have arisen at high rate by inter‐allelic recombination (peptide groove sequence targeting via HLA gene conversion events^17^, ^18^). For example, such striking events are thought to have taken place between existing founding HLA‐B and HLA‐C alleles when humans colonized the Americas by migration from eastern Asia 10 000 to 40 000 years ago.^15^ Targeted gene conversion events of this type involving the DNA sequences encoding the peptide‐binding groove sites of MHC I/II genes are well documented.^18^, ^19^, ^20^, ^21^, ^22^

Indeed, over 25 years ago Pease and associates^20^ invoked a simple mechanism to explain the emergence in the mouse germline of unusual H‐2 mutation patterns. They published H‐2 mutation data involving complex mosaic gene conversion tracts. To explain their data, they invoked the idea of an mRNA template being necessary to encode H‐2 Class I/II intermediates and reverse transcription as the most economical explanation for the complex mosaic tract patterns observed.^20^ The role for reverse transcription involving an mRNA intermediary has been previously discussed by us in SHM itself and will be discussed further below.^23^, ^24^, ^25^, ^26^, ^27^, ^28^

## 3 POSITIVE AVIDITY‐BASED TCR SELECTION AMONG THYMIC REGULATORY T CELLS

**Figure 2:**
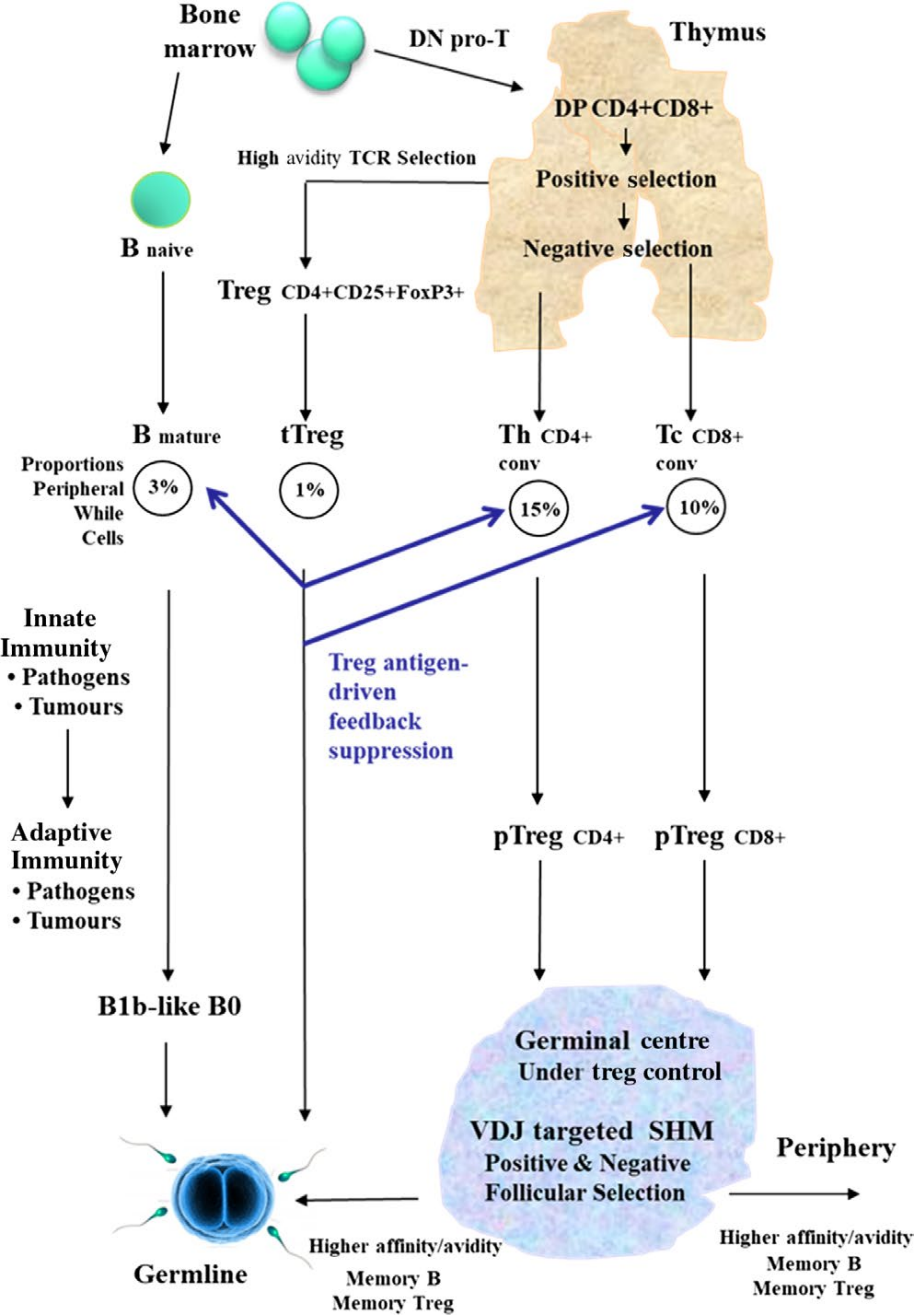
Regulatory T cells, Diversification of the αβ TCR V[D]J Somatic Repertoire, Maintenance and Co‐Evolution of Species‐Specific αβ TCR V regions and MHC alleles. Schematic outline of the key points discussed in the text. Proportions of cell types in periphery are based on Melzer et al 2015^65^

We accept the evidence for Dembic’s proposal^4^ of competitive high‐avidity TCR anti–self‐MHCI/II binding *per se* during ontogeny in the thymus. The review of that evidence by Dembic links it to subsequent migration of such positively selected thymic tTregs into the periphery where they act as immediate backup regulatory mechanisms during immune responses to non–self‐antigens – to maintain central tolerance against self T cell and B cell reactivity.^14^, ^29^ Our prediction then is these high‐avidity tTregs are also potential donors of anti–self‐MHCI/II TCR V sequences to germline V arrays prior to reproduction, thus maintaining the self/species‐specific bias in the germline TCR V repertoires (see Figure 2). That is, as with the anti‐self bias of B1b‐like B0 cells^1^ we are suggesting that a peripheral Treg cell migration pathway exists, and that it targets reproductive tissues (among others), and thus provides a gene donation (gene conversion) pathway into germ cells for the beneficial immune health and memory of the next generation. The causal links are summarized in Figure 2.

## 4 THE GERMINAL CENTRE: SITE OF BCR AND TCR RECEPTOR REVISION AND MUTATION – SELECTION

Over the past 25 years, the Germinal Centre has established itself as the main post‐antigenic site in the periphery for BCR and TCR ‘Receptor Mutation/Replacement Programs’.^30^, ^31^, ^32^, ^33^ This is generally overtly accepted for the rapid SHM of BCR Igs and thus their mutated and modified secreted antibodies. For B cells, Nemazee and Weigert^30^ succinctly summarize ‘Working together, receptor selection and clonal selection (in the GC) account for the astonishing rapidity of the somatic evolution of immune specificity’.

The rhetorical question then is ‘Why should such an important somatic evolution process be restricted *only* to B cells, and not their equally important T cell counterparts?’ In our view, the evidence for GC‐driven somatic mutation in mammalian TCR is strong,^5^, ^6^ and the objections against it are more emotional than scientific. This reflection is also coupled to our observation that very few technically careful GC‐focused studies *in vivo* have actually been published, as were executed in the 1990s by the Kelsoe^5^ and Wain‐Hobson^6^ groups. The fact that somatic hypermutation in the TCRs of lower vertebrate fish are commonplace^34^, ^35^, ^36^, ^37^ underlines our opinion here that ongoing objections to antigen‐driven GC‐mediated SHM in mammalian TCRs are not based on a sound scientific foundation.

## 5 INDUCED PERIPHERAL T REGULATOR CELLS

In the periphery, antigen stimulation and innate immune inflammatory responses can induce conventional CD4+ T cells to become peripheral CD4+CD25+ FoxP3+ pTregs 38, 39, 40, 41 and more importantly it is now clear that induced pTregs play important roles in both Germinal Centre formation and the regulation of such sites of B lymphocyte hypermutation, memory formation and affinity/avidity maturation.^42^, ^43^, ^44^, ^45^ Of more importance for our argument, here is the accumulating evidence for a key role of induced peripheral CD8+ Tregs in the Germinal Centre reaction itself.^46^, ^47^

All the indications are that the Germinal Centre‐mediated programme of RAG‐assisted ‘Receptor Mutation, Replacement, Revision’ as summed up by Nemazee and Weigert^30^ is not restricted to B cells. Thus, RAG‐assisted V replacement programmes, particularly the Ig VH segment to Ig VDH replacement process^31^, ^48^, ^49^, ^50^ and secondary V[D]J rearrangements can also be orchestrated in T cells in Germinal Centres or the immediate cellular environment.^32^, ^33^, ^51^ Many V replacement/revision events, which depend on sequence homologies to RAG‐recognized embedded heptamers at the 3’ end of framework region 3 probably go undetected.^30^, ^31^ Thus, ‘VH replacement at the downstream heptamer is essentially “invisible,” since most of the recipient gene is erased “.. a profound reason for underestimating the extent of VH editing ..” of his type.^30^

Thus, the evidence suggests that there is no reason to think that the phenomenon of TCR revision documented by Fink and associates^32^, ^33^, ^51^ is not the same, or at least very similar in molecular mechanism to, targeted RAG‐assisted VH to VHDJH replacement in B cells, as demonstrated by Weigert and associates. Indeed, the shorter N regions in TCR Vβ revisions reported by Fink and associates are suggestive of clean replacement conversions by related endogenous Vβs of the target Vβ5 segment in the VDJ rearrangement. Consequently, after TCR Vβ5 revision the ‘.. endogenous Vβ sequences in Vβ5 transgenic mice are distinguishable from similar sequences in wild type non‐transgenic controls. The former is characterized by shorter N regions than the latter ..’.^52^ One interpretation of these data is that N additions are suppressed during putative V to VDJ gene conversion replacements allowing clean V replacement (gene conversion) events to take place.

## 6 SOMATIC HYPERMUTATION IN THE MAMMALIAN TCR?

How strong then is the evidence for somatic hypermutation of mammalian TCRs? As just discussed, we believe the evidence is strong, and that the two groups which secured that evidence^5^, ^6^ are (a) highly reputable scientists, (b) were both very careful to measure PCR error rates, and (c) they audited their data for PCR recombinant artefacts (known to blunt SHM strand biases in conventional Ig VDJ GC‐mediated mutagenesis).^25^ Highly technical skills were required to do these micro‐manipulation cell samplings. Further, both groups comment on the striking similarity of the TCR mutation patterns at A:T and G:C base pairs as in conventional Ig SHM.

We are very familiar with these types of immunoglobulin diversity data having spent many years of analysis of Ig SHM patterns in B cell VDJ loci and the molecular mechanism 23, 24, 25, 26, 27, 28, 53 and we agree and concur with their conclusions. Despite the small sample sizes, the strand biases noted by both groups for mutations of A exceeding mutations at T (A>>T, as reported in Zheng et al.^5^ and mutations of G exceeding mutations of C (G>>C, as reported in Cheynier et al. 6 are the same as what is observed in Ig SHM patterns. These patterns are also observed in off‐target non‐Ig dysregulated Ig SHM‐like responses in both TP53 sequence substrates^54^, ^55^ and across cancer genomes generally.^54^, ^55^, ^56^, ^57^

However, in Cheynier et al.^6^, the low level of somatic mutations at A:T base pairs is barely above the *Taq* polymerase PCR error rate so in our view these particular mutation data are focused on G:C base pairs, thus indicative of mainly AID/APOBEC‐induced cytosine to uracil mutagenesis at their familiar WRC and related C‐centred motifs. However, the strong signal of strand bias at G:C base pairs is *exactly* what is seen and predicted by the reverse transcriptase mechanism of Ig SHM both in normal on‐target Ig substrates and when dysregulated at non‐Ig substrates in cancer genomes.^27^, ^55^, ^56^

In HIV‐1 and similar SIV infections in humans and non‐human primates CD8+ Tregs are prominent in Germinal Centres.^46^, ^47^ Such follicular CD8+ T cells are also the cells hypermutating in the white pulps of the Germinal Centres of HIV‐1 patients analysed by Cheynier et al.^6^ It is plausible then to suggest that GC‐derived CD8+ pTregs are the cellular sites of TCR‐SHM in Germinal Centres. In other work, CD4+ T cells have also been reported to express AID deaminase.^58^

## 7 HOW IS SOMA‐TO‐GERMLINE FEEDBACK OF TCR V REGIONS LIKELY TO OCCUR?

This has been discussed earlier in Steele and Lindley^1^ and Steele and Lloyd^12^ on the basis of contemporary data. The overall major steps are shown in Figure 2. The suggestion we made^1^ for B1b‐like B0 cells still holds, and is now hypothesized to be augmented by the addition of new steps that implicate the key role of tTregs and pTregs:

**Step 1**. Both high‐avidity anti–self‐MHC thymic Tregs and GC‐derived peripheral Tregs bearing mutated and affinity tested TCRs home to reproductive tissues, and as first hypothesized for hypermutated memory B cells by Rothenfluh. 59

**Step 2.** The actual soma‐to‐germline feedback (S>G) occurs primarily through TCR V[D]J retro‐transcripts targeting similar germline TCR V segments causing partial or complete V replacement gene conversion. We speculated then, and speculate again here, that enzymatic RAG‐assisted like variations of the Weigert‐Nemazee‐Fink V replacement‐editing/revision scenarios are now co‐opted, but are now operating in the reverse direction of VDJ→V , based on V sequence homologies of incoming V[D]Js with homologous V targets in the germline V segment arrays,^30^, ^51^ and see Figure 1 in Steele and Lloyd.^12^

Finally, the recent comparative sequence analysis by Watson and associates^60^ of the human IGHV locus spanning about 1 Mb at 14q32.33 is very informative of the structure of the Stage 1 VH germline repertoire, and relevant to any future germline TCR analyses. From their contiguous sequence of this region from a hydatidiform mole the Watson et al.^60^ data allow, by comparison with the earlier reference IGHV sequence of Matsuda et al.^61^ an estimate of the *actual size* of the highly conserved anti–self‐component of Cohn‐Langman’s human IGHV Stage I segment repertoire.^62^, ^63^ This is discussed by Steele and Lindley^1^ on the conclusion summarized by the data in Figure 1 of Watson et al.^60^ It can be deduced that of the functional 40‐50 odd IGHV segments about half share an unaltered VH sequence and the other half differ as alleles or novel functional VHs. Thus, about 25 human VH segments are highly conserved and most likely play a physiological role in natural antibodies in the disposal of effete self components, or in providing protection against highly predictable endemic pathogens. There are other implications of these data. However, this informative comparative sequencing strategy of Watson and associates could be equally applied to estimates of the conserved *versus* plastic component of the human germline TCR V repertoires at TCR α and β loci haplotypes. The stratification of such data by ethnicity, as well as by three‐generation pedigree analyses, would also be extremely valuable in understanding the evolution of BCR and TCR germline V repertoires.^12^

In a recent other important study, we also consider that these S>G events also occur more generally, and provide a likely explanation for the likely deaminase origin of many human SNPs in non‐Ig genes.^13^ NGS technology, particularly the new PacBio techniques of long read sequencing through highly repetitive regions, such as Ig V and TCR V arrays should now make it possible to analyse at nucleotide resolution transgenerational extended family ‘tracking’ experiments on scale, that were not previously thought possible. So germline V array sequence analyses with additional stratification of such families by levels of exposure to infectious diseases are now possible.

## 8 SUMMARY & CONCLUSIONS

We advance here a model shown schematically in Figure 2 for the co‐evolution of allele‐specific MHC recognition by the TCR confining the initial cellular events to somatic selection of thymic and peripheral Tregs. The model has grown quite naturally out of the recent proposal of Dembic^4^ and provides a mechanism to explain some previously long‐held assumptions, for example of Cohn et al.^64^, of MHC species‐specific allele recognition by the TCR. In the past, both the soma‐to‐germline feedback proposal and invocation of somatic hypermutation occurring in TCRs of mammalian T cells have been very controversial and thus inhibitory of ongoing research. We hope to defuse some of the opposition to these ideas by showing how useful they can be in building our molecular understanding of the evolution of TCR recognition of allele‐specific MHC Class I and Class II + peptide. More speculatively, it is conceivable that in the future such S>G processes might also apply to the coupled conservation and diversification of many other endogenous arrays of receptor‐ligand genetic systems.

Finally, it is worth noting that the novel proposition that Tregs within Germinal Centres are also the cellular home for AID/APOBEC and ADAR deaminase‐driven TCR‐SHM – just like Ig‐SHM in GC B cells – can now be tested experimentally. Such experiments can benefit from current high‐throughput V[D]J repertoire NGS sequencing and single‐cell sequencing technologies. However, careful attention to technical details in the cellular and somatic genetic analysis of structure and dynamics of *in vivo* GCs is still required – akin to the technical expertise displayed in those few pioneering experiments conducted by Kelsoe, Wain‐Hobson and their associates 20‐25 years ago.^5^, ^6^

## CONFLICT OF INTEREST

The authors declare no conflict of interest.
